# Endogenous Opiates in the Nucleus Tractus Solitarius Mediate Electroacupuncture-Induced Sleep Activities in Rats

**DOI:** 10.1093/ecam/nep132

**Published:** 2011-02-13

**Authors:** Chiung-Hsiang Cheng, Pei-Lu Yi, Jaung-Geng Lin, Fang-Chia Chang

**Affiliations:** ^1^Department of Veterinary Medicine, School of Veterinary Medicine, National Taiwan University, Taipei 106, Taiwan; ^2^Department of Medical Technology, Jen-Teh Junior College of Medicine, Nursing and Management, Miaoli, Taiwan; ^3^Graduate Institute of Acupuncture Science, College of Chinese Medicine, China Medical University, Taichung, Taiwan

## Abstract

Electroacupuncture (EA) possesses various therapeutic effects, including alleviation of pain, reduction of inflammation and improvement of sleep disturbance. The mechanisms of EA on sleep improvement, however, remain to be determined. It has been stated in ancient Chinese literature that the Anmian (EX17) acupoint is one of the trigger points that alleviates insomnia. We previously demonstrated that EA stimulation of Anmian acupoints in rats during the dark period enhances non-rapid eye movement (NREM) sleep, which involves the induction of cholinergic activity in the nucleus tractus solitarius (NTS). In addition to cholinergic activation of the NTS, activation of the endogenous opioidergic system may also be a mechanism by which acupuncture affects sleep. Therefore, this study was designed to investigate the involvement of the NTS opioidergic system in EA-induced alterations in sleep. Our present results indicate that EA of Anmian acupoints increased NREM sleep, but not rapid eye movement sleep, during the dark period in rats. This enhancement in NREM sleep was dose-dependently blocked by microinjection of opioid receptor antagonist, naloxone, and the *μ*-opioid receptor antagonist, naloxonazine, into the NTS; administrations of *δ*-receptor antagonist, natrindole, and the *κ*-receptor antagonist, *nor*-binaltrophimine, however, did not affect EA-induced alterations in sleep. Furthermore, *β*-endorphin was significantly increased in both the brainstem and hippocampus after the EA stimuli, an effect blocked by administration of the muscarinic antagonist scopolamine into the NTS. Our findings suggest that mechanisms of EA-induced NREM sleep enhancement may be mediated, in part, by cholinergic activation, stimulation of the opiodergic neurons to increase the concentrations of *β*-endorphin and the involvement of the *μ*-opioid receptors.

## 1. Introduction

Electroacupuncture (EA), which consists of passing a continuous electric current through needles inserted into the acupoints to obtain the therapeutic effects, that is, alleviation of pain, reduction of inflammation and management of insomnia, is modified from the traditional Chinese acupuncture. Insomnia is one of the most common sleep disorders and it has been demonstrated that the effectiveness rate of acupuncture for relieving insomnia is about 90% [1, 2]. Several specific acupoints have been identified for insomnia treatment based upon the differentiation and signs of symptoms according to traditional Chinese medicine. Among the acupoints used, Shenmen (HT7), Sanyinjiao (SP6) and Anmian (EX17) are the most common, although other acupoints may also be used, such as Neiguan (PC6), Zusanli (ST36), Taichong (LR3), Baihui (DU20), Dazhui (DU40), Tainzhu (BL10), Bishu (BL20) and Zhongwan (RN12) [3, 4].

The mechanisms by which EA functions to alleviate clinical symptoms remain largely unknown, although applications of EA have been widely described in the Chinese literature. The spinal gate-control theory [5] and the activation of central endorphin and/or monoaminergic systems (i.e., serotonin and norepinephrine) [6] have been hypothesized in mediating the EA-induced analgesia. In addition, acupuncture may reduce the inflammation-induced elevation of body temperature by suppressing hypothalamic production of pro-inflammatory cytokines [7]. The central opioidergic and serotonergic systems also mediate the suppressive effects of acupuncture on capsaicin-induced neurogenic inflammation [8]. Recent findings suggest that the induction of vagus nerve activity appears to be another significant factor for mediating the action of acupuncture [9, 10]. The caudal nucleus tractus solitarius (NTS) may be activated by acupuncture, since NTS is located in the dorsomedial medulla oblongata and receives afferents primarily from the vagus and glossopharyngeal nerves [11]. Ascending projections from the NTS are traced through the lateral and dorsal tegmentum and periventricular gray up to the rostral pons and midbrain, and terminate in the parabrachial nucleus, which in turn projects to the thalamus, hypothalamus, preoptic area, bed nucleus of the stria terminalis, amygdala and the frontal cortex, regions commonly belonging to the visceral-limbic forebrain [12, 13]. From these anatomical data, it does not appear that the predominant effect of the NTS is via the reticular activating system but instead is via limbic forebrain structures, which are implicated in the sleep regulation. Furthermore, the low-frequency electrical stimulation of the medullary reticular formation, particularly the dorsal reticular formation and the caudal NTS, produces cortical synchronization indicative of slow-wave sleep (SWS) in an awake animal [14]. Conversely, lesions of the dorsal reticular formation and of the NTS produced desynchronization of the EEG in a sleeping animal [15]. These results all suggest the existence of neurons in the NTS that are involved in generating sleep. Furthermore, microinjection of morphine into the NTS provokes an enhancement of SWS and this effect is blocked by naloxone [16], suggesting the somnogenic effect of opioidergic system in the NTS. Our previous observations demonstrate that activation of cholinergic system in the caudal NTS of the medulla oblongata mediates the enhancement of non-rapid eye movement (NREM) sleep induced by EA stimulation of Anmian (EX17) acupoints [17]. Nonetheless, EA may also increase *β*-endorphin concentrations in the NTS, which subsequently alter sleep, because the NTS area is one of the anatomically distinct *β*-endorphin pathways in the brain influenced by EA [18]. Therefore, this study was designed to further clarify whether the activation of cholinergic system in the NTS after EA stimuli enhances endogenous opioidergic activity and what type(s) of opioid receptors are involved in EA-induced alterations in sleep.

## 2. Methods

### 2.1. Pharmacological Agents

Stock solutions of muscarinic receptor antagonist, scopolamine hydrobromide (Sigma, St Louis, MO, USA), a broad-spectrum opioid antagonist (naloxone hydrochloride (Tocris, Bristol, UK)), a *μ*-receptor antagonist (naloxonazine dihydrochloride (Tocris)), a *δ*-receptor antagonist (naltrindole hydrochloride (Tocris)) and a *κ*-receptor antagonist (*nor*-binaltorphimine dihydrochloride (Tocris)) were dissolved in pyrogen-free saline (PFS). The stock solutions were stored at 4°C until use. The dose of scopolamine used was 20 *μ*g *μ*L^−1^, whereas naloxone, naloxonazine, naltrindole and *nor*-binaltorphimine were microinjected at three different doses, 0.1, 1.0 and 10 *μ*g *μ*L^−1^. The total volume used for each microinjection was 1 *μ*L.

### 2.2. Animals

Male Sprague-Dawley rats (250–300 g; National Laboratory Animal Breeding and Research Center, Taiwan) were used in this study. Rats were anesthetized by intraperitoneal injection with ketamine/xylazine (87/13 mg kg^−1^) and were given an analgesic (1 mg/rat morphine) and an antibiotic (5000 IU/rat penicillin G benzathine) to reduce pain and avoid infection. Rats were surgically implanted with three electroencephalogram (EEG) screw electrodes as earlier described [19] and the microinjection guide cannulae directed into the NTS (AP, 13.30 mm from bregma; ML, 1.2 mm and DV, 8.2 mm relative to bregma). The coordinates were adopted from the Paxinos and Waton rat altas [20]. Two unilateral screw EEG electrodes were placed over the right hemisphere of the frontal and parietal cortices and a third EEG electrode was placed over the cerebellum and served to ground the animal to reduce signal artifacts. Insulated leads from EEG electrodes were routed to a Teflon pedestal (Plastics One, Roanoke, VA, USA). The Teflon pedestal was then cemented to the skull with dental acrylic (Tempron, GC Co., Tokyo, Japan). The incision was treated topically with polysporin (polymixin B sulfate—bacitracin zinc) and the animals were allowed to recover for 7 days prior to the initiation of experiments. The rats were housed separately in individual recording cages in the isolated room, in which the temperature was maintained at 23  ±  1°C and the light:dark rhythm was controlled in a 12 : 12 h cycle (40 Watt × 4 tubes illumination). Food (5001 rodent diet, LabDiet) and water were available *ad libitum*. All procedures performed in this study were approved by the National Taiwan University Animal Care and Use Committee.

### 2.3. Experimental Protocol

On the second postsurgical day, the rats were connected to the recording apparatus via a flexible tether. As such, the rats were allowed relatively unrestricted movement within their own cages. Three groups of rats were used in the study as follows: Group 1 (*n* = 8) was used to determine the effects of opioid receptor antagonist (naloxone) and *μ*-receptor antagonist (naloxonazine) on EA-induced alterations in sleep; Group 2 (*n* = 8) was used to elucidate the effects of *δ*-receptor antagonist (naltrindole) and *κ*-receptor antagonist (*nor*-binaltorphimine) on EA-induced alterations in sleep; Group 3 (*n* = 24) was used to depict the action of NTS muscarinic receptors on EA-induced *β*-endorphin expression (determined by enzyme-linked immunosorbent assay (ELISA)) after microinjection of scopolamine into the NTS. One week after rats had adapted to the 12 : 12 h light : dark cycle after surgery, a 23-h undisturbed baseline recordings were obtained beginning at dark onset on the first recording day in rats in Groups 1 and 2. When EA was given (see later), all rats were lightly anesthetized with one-third of the dose of ketamine/xylazine used in the surgery, after which rat wake-up time is 20–25 min. A 20-min period of EA stimulation was administered before the onset of the dark period. The anesthetization was given 25 min prior to the dark period onset and lasted for 20 min. The rationale for carrying out the experiment in the darkness is that rats are active with a lowest level of sleep during the dark period, and a manipulation, if it possesses ability to increase sleep, would significantly augment sleep during the dark period. In contrast, it may not be easy to enhance sleep during the light period when sleep activity is at its highest circadian level. Since we expected to find a sleep enhancement after the EA stimuli at the Anmian (EX17), we therefore manipulated the EA stimulation before the onset of the dark period and analyzed the sleep alteration during the subsequent dark period. The rats in group 1 were both administered PFS intraperitoneally (IP) and microinjected into the NTS (_ip_PFS + PFS) at 25 min prior to the dark onset on two consecutive days, and recordings obtained for 24-h beginning after the second injection. The effects of anesthesia with the NTS microinjection of PFS (_ip_ketamine + PFS) on sleep were determined after IP injection of ketamine/xylazine and the NTS PFS microinjection on two consecutive days. A sham EA (_ip_ketamine + PFS + sham EA) was delivered to control for the non-specific effect of the electrical stimulation, although our previous study had confirmed that no non-specific effect was observed after the sham EA [17]. The EA stimuli under anesthesia (_ip_ketamine + PFS + EA) were also performed before the dark onset on two consecutive days and sleep-wake behavior after the second EA stimulation was then determined. Subsequently, three different doses (0.1, 1.0 and 10 *μ*g) of naloxone (_ip_ketamine + naloxone + EA) and naloxonazine (_ip_ketamine + naloxonazine + EA) were administered 25 min prior to the dark onset on the second day of EA stimulation and the 24-h sleep pattern was determined. At least 1 day without injections was scheduled between each manipulation. The EA stimulus was delivered via the bilateral insertion of stainless needles (32 gauge × 1^*″*^, Shanghai Yanglong Medical Articles Co.) on Anmian (EX17) points in the depth of 2 mm. The stimulus consisted of a train of biphasic pulses (150 *μ*s duration each) of 10 Hz with intensity of 3 mA, and was delivered by Functions Electrical Stimulator (Trio 300, I.T.O., Japan). The acupoint “Anmian (EX17)” is located at midpoint between Yifeng (TH 17) and Fengchi (GB 20); Yifeng (TH 17) locates posterior to the lobule of the ear in the depression between the mandible and mastoid process and Fengchi (GB 20) locates in the depression between the upper portion of musculus sternocleidomastoideus and musculus trapezius in human. The location of Anmian (EX17) in rats is at the relative anatomical location between the strenocleidomastoideus muscle and the splenius capitis muscle, as in the human acupoint map. Sham EA was performed by stimulation of a non-acupoint located at the ventral conjunction between the forelimb and the trunk as described earlier [17]. Those rats in group 2 underwent a similar protocol to those in group 1, except that the substances administered were naltrindole (_ip_ketamine + naltrindole + EA) and *nor*-binaltorphimine (_ip_ketamine +* nor*-binaltorphimine + EA). Rats in group 3 were divided into four subgroups and received _ip_ketamine + PFS, _ip_ketamine + PFS + sham EA, _ip_ketamine + PFS + EA and _ip_ketamine + scopolamine + EA, respectively. Microinjection of scopolamine into the NTS was administered prior to the second EA stimulation. Rats were then decapitated at the dark onset and five distinct brain regions, including the hypothalamus, cortex, brainstem, hippocampus and striatum, were dissected and frozen in −80°C until assay. These tissues were used for *β*-endorphin ELISA as described in the following.

### 2.4. Apparatus and Recording

Signals from the EEG electrodes were fed into an amplifier (Colbourn Instruments, Lehigh Valley, PA; model V75-01). The EEG was amplified (factor of 5000) and analog bandpass filtered between 0.1 and 40 Hz (frequency response: ±3 dB; filter frequency roll off: 12 dB/octave). Gross body movements were detected by custom-made infrared-based motion detectors (Biobserve GmbH, Germany) and the movement activity was converted to a voltage output which was digitized and integrated into 1-s bins. These conditioned signals (EEGs and gross body movements) were subjected to analog-to-digital conversion with 16-bit precision at a sampling rate of 128 Hz (NI PCI-6033E; National Instruments, Austin, TX). The digitized EEG waveform and integrated values for body movement were stored as binary computer files pending subsequent analyses.

Postacquisition determination of the vigilance state was done by visual scoring of 12-s epochs using custom software (ICELUS, M. R. Opp) written in LabView for Windows (National Instruments). The animal's behavior was classified as SWS, rapid eye movement sleep (REMS) or waking based on previously defined criteria [19]. Briefly, SWS is characterized by large-amplitude EEG slow waves, high power density values in the delta frequency band (0.5–4 Hz) and lack of gross body movements. During REMS, the amplitude of the EEG is reduced, the predominant EEG power density occurs within the theta frequency (6–90 Hz) and there are phasic body twitches. During waking, the rats are generally active. There are protracted body movements. The amplitude of the EEG is similar to that observed during REMS, but power density values in the delta frequency band are generally greater than those in theta frequency band. In addition to the amount of time spent in each vigilance state, the number and duration of individual bouts of behaviors were determined using criteria modified from those of Tobler and colleagues [21, 22], as described earlier [19].

### 2.5. ELISA for *β*-Endorphin

Rat *β*-endorphin ELISA kits were obtained from Phoenix Pharmaceuticals Inc. (Burlingame, CA, USA) and the procedures followed the standard instructions provided by the manufacturers. Absorbance was measured by an ELISA plate reader (Multiskan EX, Thermo Electron Corp., Waltham, MA, USA) with the wavelength set at 450 and 550 nm. The sensitivity is 0.21 ng mL^−1^, the assay range is between 0.21 and 3.15 ng mL^−1^, the intra-assay coefficient of variation is <5% and the inter-assay coefficient of variation is <14%. There is no cross-reactivity for met-enkephalin and leu-enkephalin.

### 2.6. Statistical Analyses for Experiment Protocol

All values acquired from sleep-wake recording were presented as the mean  ±  SEM for the indicated sample sizes. One-way analyses of variance (ANOVA) for the duration of each vigilance state (SWS, REMS, WAKE) and for sleep architecture parameters were performed, comparing before and after manipulation within subjects, across a certain of time block. An *α* level of *P* ≤ .05 was taken as indicating a statistically significant difference. If statistically significant differences were detected, a *Scheffe post hoc* comparison was made to determine which hourly intervals during experimental conditions deviated from values obtained from the same animals during control conditions. The statistical evaluation for the *β*-endorphin ELISA used an unpaired Student's *t*-test comparing the averages between the groups analyzed.

## 3. Results

### 3.1. Naloxone and Naloxonazine Blocked the EA-Induced Alterations in Sleep

**Figure 1:**
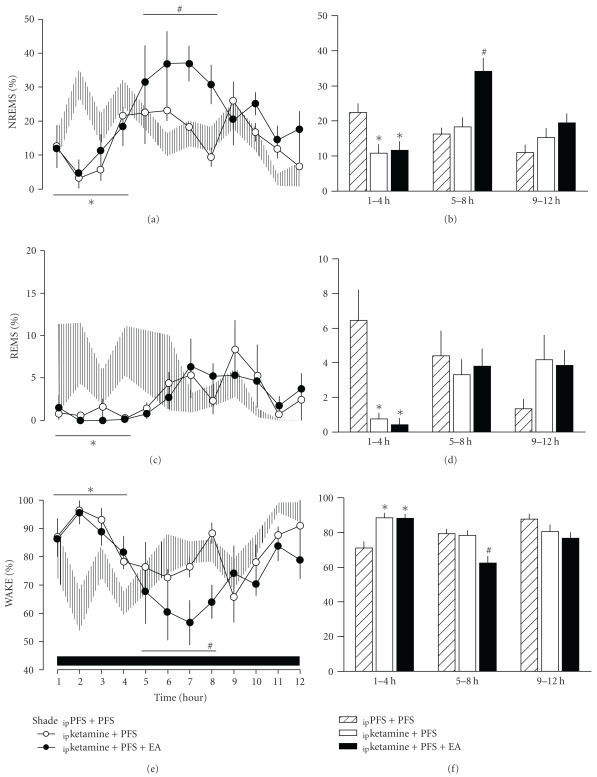
The effects of low-frequency (10 Hz) stimulation by EA at Anmian (EX17) acupoints on vigilance states. The 10 Hz EA enhanced NREM sleep during post-manipulation 5–8 h during the subsequent dark period. *Represents a statistically significant difference between the values obtained from _ip_ketamine + PFS and _ip_PFS + PFS. The shaded area represents the values of mean  ±  SEM. ^#^Depicts a statistically significant difference between the values obtained from _ip_ketamine + PFS + EA and _ip_ketamine + PFS. The black horizontal bar on the *x*-axis represents the dark period of the 12 : 12 h light : dark cycle. WAKE, wakefulness.

Anesthetization of rats for 25 min with ketamine/xylazine prior to the dark period suppressed both NREM and REM sleep for the first 4 h of the dark period. The time spent in NREM sleep during the first 4-h period after _ip_ketamine + PFS decreased to 10.8  ±  2.5% from 22.4  ±  2.5% acquired after _ip_PFS + PFS (*F*_(1,62)_ = 10.711, *P* = .002, Figures 1(a) and 1(b)). REM sleep was suppressed from 6.5  ±  1.8% obtained after _ip_PFS + PFS to 0.8  ±  0.3% acquired after _ip_ketamine + PFS (*F*_(1,62)_ = 8.697, *P* = .005, Figures 1(c) and 1(d)). A concomitant enhancement of wakefulness was observed during the first 4 h after receiving ketamine anesthetization (Figures 1(e) and 1(f)). Application of sham EA did not alter any aspect of sleep parameters (data not shown), which is similar to our previous observation [17]. Twenty-minute EA stimuli delivered before the dark period on two consecutive days significantly augmented NREM sleep during post-manipulation 5–8 h. The percentage of time spent in NREM sleep during 5–8 h increased from 18.3  ±  2.7% obtained after _ip_ketamine + PFS to 34.0  ±  3.8% after vketamine + PFS + EA (*F*_(1,62)_ = 9.180, *P* = .004, Figures 1(a) and 1(b)). Analysis of 12-h dark period revealed that NREM sleep was enhanced from 14.8  ±  1.5% after _ip_ketamine + PFS to 21.7  ±  2.1% after _ip_ketamine + PFS + EA (*F*_(1, 190)_ = 6.923, *P* = .010). REM sleep was not significantly altered by EA. A decrease of waking at the expense of SWS was observed during 5–8 h (Figures 1(e) and 1(f)).

**Figure 2:**
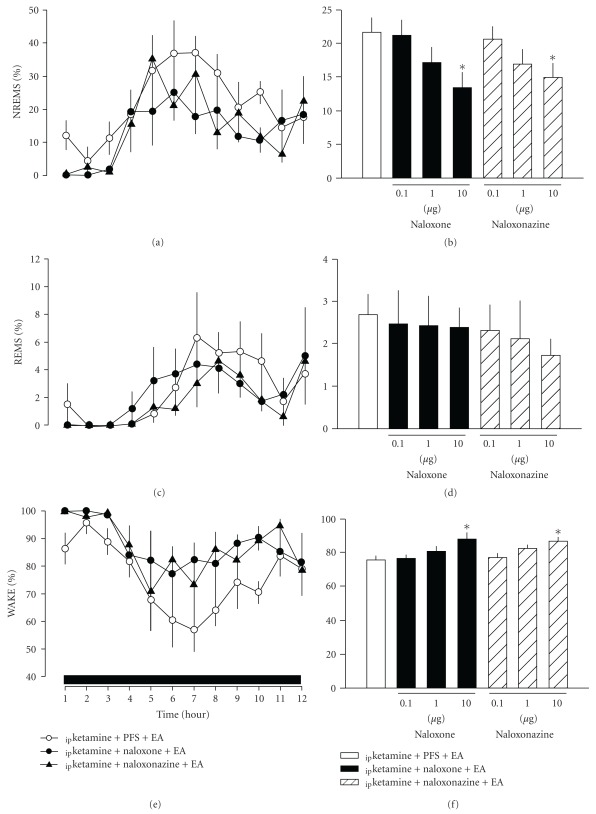
The effects of naloxone and naloxonazine on EA-induced alterations in sleep. Both naloxone and naloxonazine dose-dependently blocked the EA-induced enhancement of NREM sleep. *Represents a statistically significant difference between the values obtained from _ip_ketamine + naloxone + EA/_ip_ketamine + naloxonazine + EA and _ip_ketamine + PFS + EA. The black horizontal bar on the *x*-axis represent the dark period of the 12 : 12 h light : dark cycle.

Administration of three different doses (0.1, 1.0 and 10.0 *μ*g) of naloxone, a broad-spectrum opioid antagonist, into the NTS dose-dependently blocked EA-induced increases of NREM sleep, especially during post-manipulation 5–8 h (Figures 2(a) and 2(b)). Across the entire 12-h dark period recording, NREM sleep was suppressed from 21.7  ±  2.1% after _ip_ketamine + PFS + EA to 13.4  ±  2.1% after _ip_ketamine + naloxone (10.0 *μ*g) + EA (*F*_(1,190)_ = 7.631, *P* = .007, Figures 2(a) and 2(b)). Administration of *μ*-opioid receptor antagonist, naloxonazine, also exhibited a dose-dependent blockade of EA-induced NREM sleep enhancement (Figures 2(a) and 2(b)). Administration of 10 *μ*g naloxonazine reduced NREM sleep from 21.7  ±  2.1% after _ip_ketamine + PFS + EA to 14.9  ±  2.1% (*F*_(1,190)_ = 5.100, *P* = .026, Figures 2(a) and 2(b)) during the 12-h dark period. Neither naloxone nor naloxonazine altered REM sleep (Figures 2(c) and 2(d)). A mirror effect on the EA-induced decrease of waking was also been observed for both naloxone and naloxonazine (Figures 2(e) and 2(f)).

Analysis of sleep architecture parameters across 1–12 h during the dark period revealed that the effect of ketamine on the suppression of NREM and REM sleep was primarily due to an increase in episode duration of waking, although there was a tendency for decreases in both REM sleep episode number and in NREM sleep episode duration (Table 1). The increase of NREM sleep after EA stimuli was primarily because of the increase in the duration of a single episode (Table 1), which is similar to our previous result [17]. Effects of naloxone and naloxonazine on blocking EA-induced enhancement of NREM sleep were mediated by reversing the EA-induced augmentation of NREM sleep episode duration (Table 1).

### 3.2. Naltrindole and Nor-Binaltorphimine Did Not Affect EA-Induced Alterations in Sleep

**Figure 3:**
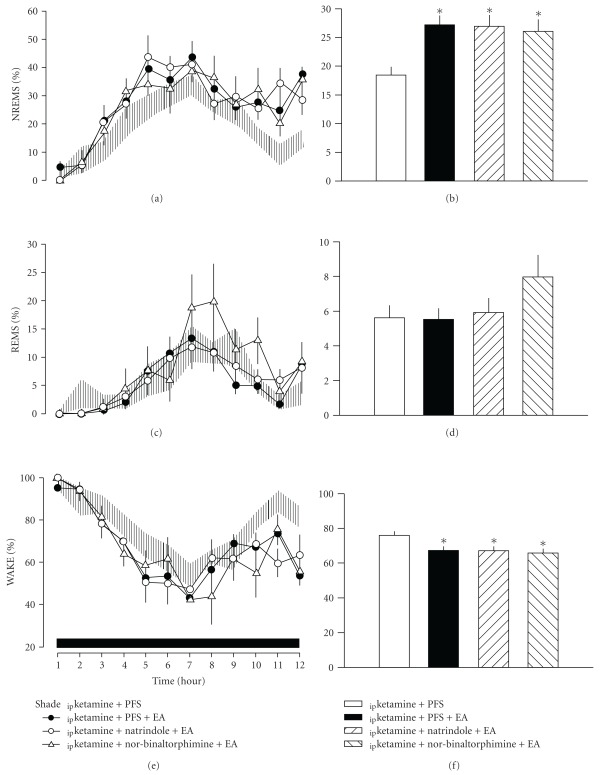
The effects of naltrindole and *nor*-binaltorphimine on EA-induced alterations in sleep. Neither naltrindole nor *nor*-binaltorphimine affected EA-induced enhancement of NREM sleep. *Represents a statistically significant difference between the values obtained from _ip_ketamine + PFS + EA/_ip_ketamine + natrindole + EA/_ip_ketamine  + *nor*-binaltorphimine + EA and _ip_ketamine + PFS. The shaded area represents the values of mean  ±  SEM. The black horizontal bar on the *x*-axis represents the dark period of the 12 : 12 h light : dark cycle.

Administration of either the *δ*-opioid receptor antagonist naltrindole or the *κ*-receptor antagonist *nor*-binaltorphimine prior to EA stimulation did not affect EA-induced alterations in sleep (Figure 3). Similarly, these antagonists did not alter any aspect of sleep architecture (data not shown).

### 3.3. Scopolamine Suppressed EA-Induced Expression of Endogenous *β*-Endorphin

**Figure 4:**
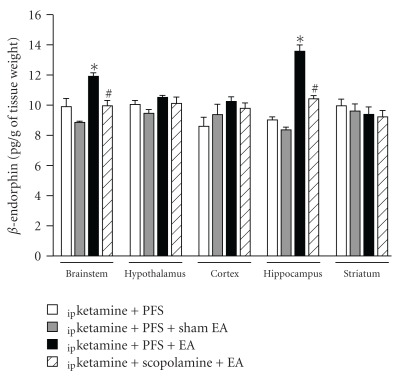
The effects of scopolamine on the 10 Hz EA stimuli-induced increase of *β*-endorphin. The 10 Hz EA stimuli significantly increased the expressions of *β*-endorphin in the brainstem and hippocampus and administration of scopolamine blocked EA-induced increases of *β*-endorphin concentration. *Represents a statistically significant difference between the values obtained from _ip_ketamine + PFS + EA and _ip_ketamine + PFS + sham EA. ^#^Depicts a statistically significant difference between the values obtained from _ip_ketamine + scopolamine + EA and _ip_ketamine + PFS + EA.

Our result demonstrated that delivery of sham EA stimuli did not alter the concentrations of *β*-endorphin within five distinct brain structures, including the brainstem, hypothalamus, cortex, hippocampus and striatum (Figure 4). However, application of EA stimuli at Anmian (EX17) acupoints for 20 min significantly enhanced the levels of *β*-endorphin in the brainstem and hippocampus, but not in the cortex, hypothalamus and striatum (Figure 4). Administration of the muscarinic receptor antagonist scopolamine directly into the NTS before the EA stimuli blocked EA-induced increases of *β*-endorphin in the brainstem and hippocampus (Figure 4).

## 4. Discussion

Insomnia is a common sleep complaint among elderly people and young adults, which may result from psychiatric illness, sociopsychological stress, a medical problem, poor sleep habits or a primary sleep disorder. Epidemiological surveys have shown that 10%–20% of adults have suffered from moderate to severe insomnia [23], although the percentage is lower than that of 40%–70% of healthy elderly people who suffer from chronic sleep disturbances [24]. Sedative-hypnotic medications, including benzodiazepines and non-benzodiazepines, are the most common treatments for insomnia. However, there are concerns regarding the inappropriate use, dependence and adverse effects of these agents. On the other hand, acupuncture has been used for relieving sleep disturbances for thousands of years in China. Although acupuncture is efficacious for sleep problems, especially for insomnia, the underlying mechanisms whereby sleep is improved by acupuncture are poorly understood. We, therefore, designed this study to determine whether the opioidergic system of the NTS plays a role in EA-induced alterations in sleep. Our current results demonstrate that EA stimuli of Anmian (EX17) acupoints in anesthetized rats for 20 min enhances NREM sleep during the subsequent dark (active) period. This observation is a further confirmation of the ability of EA stimuli at Anmian (EX17) acupoints to increase NREM sleep. In order to perform the EA stimulation easily, rats were lightly anesthetized. We found that both NREM and REM sleep during the first 4 h of the dark period were decreased after rats recovered from the ketamine anesthetic. Ketamine, a cyclohexanone derivative, is used clinically as a dissociative anesthetic agent both in humans and animals. Ketamine is a non-competitive *N*-methyl-_D_-aspartate (NMDA) receptor antagonist that blocks cation channels [25]. It has been demonstrated that administration of ketamine or MK-801, another NMDA receptor antagonist, at sub-anesthetic doses produce a robust, dose-dependent increase in the intensity of *δ*-power of the NREM sleep [26, 27]. Furthermore, the effect of MK-801 by increasing the metabolic rate in the hippocampus and other limbic structures stimulates physiological sleep that is similar to the sleep that follows sleep deprivation, indicating the need of homeostatic recovery [28]. Therefore, the suppression of NREM and REM sleep after recovery from the ketamine anesthetization during the beginning of the dark period may be due to a homeostatic compensation to the previous anesthetic state. However, this explanation needs to be further investigated. On the other hand, EA of other acupoints may exhibit an opposite effect on sleep or anesthesia. For example, EA stimulation of bilateral Neiguan (PC6) shortened the post-anesthesia recovery time in cats [29]. However, EA of Anmian (EX17) did not exhibit this effect in our experiment.

We previously demonstrated that microinjections of muscarinic receptor antagonist scopolamine into the NTS and bilateral lesions of the caudal NTS blocks the alterations in sleep induced by EA stimulation of Anmian acupoints [17], implicating the involvement of cholinergic neurons in the caudal NTS in this response. Nevertheless, endogenous opiates (*β*-endorphin, enkepalin, endomorphin and dynorphin) are well known to mediate the analgesic effect of EA; low frequency EA stimulation activates *μ*- and *δ*-opioid receptors by the releases of *β*-endorphin, enkepalin and endomorphin, whereas high-frequency EA stimulation activates the *κ*-receptors by enhancing the concentrations of dynorphin [30, 31]. It has also been demonstrated that EA increases *β*-endorphin concentrations in the arcuate nucleus of the hypothalamus via A*β* and A*δ* fibers, which in turn mediate the analgesic effects of EA [32].

There are two anatomically distinct *β*-endorphin pathways in the brain; the major pathway originates in the arcuate nucleus and the minor one is in the area of the NTS of the caudal medulla [18]. Therefore, it is possibility that *β*-endorphin concentrations may increase in the caudal NTS after the EA stimulation. Reinoso-Barbero and de Andres have shown that microinjection of morphine into the NTS provokes a dose-dependent enhancement of the polygraphic and behavioral manifestations of SWS. This effect is blocked by naloxone, suggesting that the endogenous opioid is involved in controlling electrocortical activity generated by the NTS [16]. Furthermore, it has been documented that vagal afferents activate the *β*-endorphin system in the NTS, which in turn mediates responses to vagal activation; the effects of acetylcholine on depressive responses in pulmonary and carotid arteries are mediated by activating the serotonergic and endorphin system of the NTS [33] and the inhibitory effect of vagal afferents on the bradycardia response is mediated by *β*-endorphinergic neurons in the NTS [9]. On the basis of our previous results [17] and aforementioned evidence, we herein tried to elucidate whether the effects of EA on sleep regulation is mediated by the increase of endogenous opiates and the activation of opioidergic receptors in the caudal NTS via muscarinic receptors. Application of naloxone, a broad spectrum of opioid receptor antagonist, was used to determine the involvement of NTS opioidergic receptors in the EA-induced increase of NREM sleep. We found that administration of naloxone directly into the caudal NTS dose-dependently blocked EA-induced NREM sleep enhancement during the dark period, but had no effect on the REM sleep, implicating the involvement of endogenous opiates in the NTS. A potential role for three major opioid receptors, *μ*-, *δ*- and *κ*-receptors, was then determined by application of specific receptor antagonists. Our results demonstrate that naloxonazine, a *μ*-opioid receptor antagonist, exhibits a similar dose-dependent effect as that of naloxone, whereas naltrindole (a *δ*-opioid receptor antagonist) and *nor*-binaltorphimine (a *κ*-opioid receptor antagonist) have no effect. These results are in part consistent with the results reported by Reinoso-Barbero and de Andres that administration of *μ*-and *δ*-opioid receptor agonists, but not *κ*-opioid receptor agonist, into the NTS in cats enhances NREM sleep and that REM sleep is unchanged after NTS administration of the different opioids [16]. Nevertheless, our results implicate NTS *μ*-opioid receptors as mediators of EA-induced NREM sleep enhancement, which is similar to the effect of *μ*-opioid receptors on analgesia induced by low-frequency EA stimuli. The frequency of EA we used in this study was 10 Hz, which is considered a low-frequency stimulation. Our results also indicate that concentrations of *β*-endorphin in the brainstem and hippocampus increase after EA stimuli, suggesting the involvement of NTS *β*-endorphin. The enhancement of *β*-endorphin in the hippocampus may be due to actions of multisynaptic relays from the NTS to the hippocampus [34]. Enkephalin- and *β*-endorphin-containing neurons are in the NTS [35]. Therefore, the expression of enkephalin and endorphin after low-frequency EA stimuli and the expression of dynorphin after high-frequency EA would be of interest for further investigation. Nevertheless, EA-enhanced *β*-endorphin in the NTS is blocked by microinjection of muscarinic receptor antagonist scopolamine, suggesting that the activation of endogenous opioidergic system is mediated by the NTS cholinergic nervous system.

**Figure 5:**
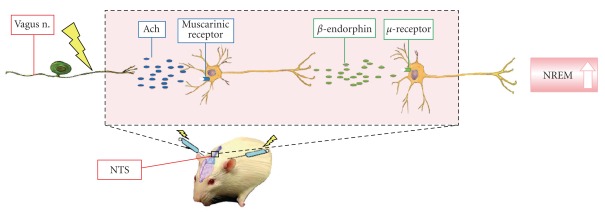
A hypothetical model by which EA Anmian (EX17) may alter NREM sleep. EA stimuli in the figure represents the electrical sign; Ach: acetylcholine; electrical sign: EA stimuli.

In summary, our current results demonstrate that EA stimuli of Anmian (EX17) acupoints enhance NREM sleep and this enhancement is blocked by naloxone and naloxonazine, implicating *μ*-opioid receptors. Furthermore, the activation of NTS muscrainic receptors after low-frequency EA stimuli increases concentrations of *β*-endorphin, which mediates the enhancement of NREM sleep after EA stimuli of Anmian (EX17) acupoints. A diagram elucidating one hypothetical mechanism by which EA of Anmian (EX17) alters sleep is depicted in Figure 5.

## Funding

National Science Council grant NSC95-2320-B-002-098-MY2.

## Figures and Tables

**Table 1:** Effects of naloxone and naloxonazine on the alterations of sleep-wake architecture parameters induced by EAc of Anmain (EX17) acupoints in rats.

Manipulation^d^	Hour	L : D cycle^e^	Number of bouts^a^			Bout duration^b^			Transitions^c^
			WAKE^f^	NREMS^f^	REMS^f^	WAKE	NREMS	REMS	
_ ip_PFS + PFS	1–12	D	3.98 ± 0.31	5.06 ± 0.45	1.58 ± 0.26	18.54 ± 2.20	1.67 ± 0.15	0.67 ± 0.10	26.94 ± 2.31
_ ip_Ketamine + PFS	1–12	D	3.52 ± 0.36	4.40 ± 0.46	1.20 ± 0.24	24.12 ± 2.97*	1.44 ± 0.15	0.59 ± 0.10	25.93 ± 2.62
_ ip_Ketamine + PFS + EAc	1–12	D	3.43 ± 0.30	4.75 ± 0.46	1.38 ± 0.24	20.57 ± 2.65	2.13 ± 0.20^#^	0.54 ± 0.09	28.81 ± 2.60
_ ip_Ketamine + naloxone + EAc	1–12	D	3.20 ± 0.44	3.48 ± 0.58	1.21 ± 0.26	29.17 ± 3.53^$^	1.15 ± 0.20^$^	0.66 ± 0.11	28.22 ± 3.51
_ ip_Ketamine + naloxonazine + EAc	1–12	D	3.19 ± 0.43	4.27 ± 0.65	0.98 ± 0.21^$^	26.16 ± 3.30	1.26 ± 0.17^$^	0.48 ± 0.10	27.93 ± 3.53

Values are means  ±  SEM.

^a^ Number of bouts per hour (mean  ±  SEM) for each vigilance state; ^b^Mean (±SEM) bout duration (min) for each vigilance state; ^c^Number of transitions from one behavioral state to another (mean  ±  SEM) per hour; ^d^Experimental manipulation; ^e^Period of the light : dark cycle immediately prior to which injections were given: D = dark period; ^f^Vigilance states: WAKE, wakefulness; NREMS, non-rapid eye movement sleep; REMS, rapid eye movements sleep; *Denotes a statistically significant difference (*P* < .05) between values obtained after administration of _ip_PFS + PFS and those obtained after receiving _ip_ketamine + PFS; ^#^Depicts a statistically significant difference (*P* < .05) between values obtained after _ip_ketamine + PFS + EAc and those obtained after _ip_ketamine + PFS; ^$^Depicts a statistically significant difference (*P* < .05) after either receiving naloxone or naloxonazine.
